# Study Protocol of a Multicenter Randomized Controlled Trial to Tackle Obesity through a Mediterranean Diet vs. a Traditional Low-Fat Diet in Adolescents: The MED4Youth Study

**DOI:** 10.3390/ijerph18094841

**Published:** 2021-05-01

**Authors:** Noemi Boqué, Lucía Tarro, Alice Rosi, Helena Torrell, Guillermo Saldaña, Elisa Luengo, Zeev Rachman, António Pires, Nuno Tiago Tavares, Ana Salomé Pires, Maria Filomena Botelho, Pedro Mena, Francesca Scazzina, Daniele Del Rio, Antoni Caimari

**Affiliations:** 1 Eurecat, Centre Tecnològic de Catalunya, Technological Unit of Nutrition and Health, 43204 Reus, Spain; noemi.boque@eurecat.org (N.B.); luciatarro@gmail.com (L.T.); 2 Human Nutrition Unit, Department of Food and Drug, University of Parma, 43125 Parma, Italy; alice.rosi@unipr.it (A.R.); pedromiguel.menaparreno@unipr.it (P.M.); francesca.scazzina@unipr.it (F.S.); daniele.delrio@unipr.it (D.D.R.); 3 Eurecat, Centre Tecnològic de Catalunya, Centre for Omic Sciences (Joint Unit Eurecat-Universitat Rovira i Virgili), Unique Scientific and Technical Infrastructures (ICTS), 43204 Reus, Spain; helena.torrell@eurecat.org; 4 NOVAPAN S.L, Research & Development Department, 50014 Zaragoza, Spain; investigacion@panishop.com (G.S.); desarrollo@panishop.com (E.L.); 5 SHIKMA Field Crops, 8531500 Kibbutz Mishmar HaNegev, Israel; zeevrach@gmail.com; 6 Pediatric Cardiology Department, Hospital Pediátrico de Coimbra, Centro Hospitalar e Universitário de Coimbra (CHUC), 3000-075 Coimbra, Portugal; antonio.pires@chuc.min-saude.pt; 7 Institute of Biophysics, Faculty of Medicine, Institute for Clinical and Biomedical Research (iCBR) Area of CIMAGO, University of Coimbra, 3000-548 Coimbra, Portugal; nuno.tiago.tavares.last@gmail.com (N.T.T.); pireslourenco@uc.pt (A.S.P.); mfbotelho@fmed.uc.pt (M.F.B.); 8 Centre for Inovative Biomedicine and Biotechnology, University of Coimbra, 3000-548 Coimbra, Portugal; 9 Clinical Academic Center of Coimbra (CACC), 3000-548 Coimbra, Portugal; 10 Eurecat, Centre Tecnològic de Catalunya, Biotechnology Area, Avinguda Universitat, 1, 43204 Reus, Spain

**Keywords:** adolescents, youth obesity, Mediterranean diet, cardiovascular risk factors, educational approach, healthy food products, omics, gut microbiota

## Abstract

Youth obesity is a strong predictor of adult obesity, which has well-known negative health consequences. Thus, addressing adult obesity requires tackling youth obesity. MED4Youth’s main objective is to strengthen the link between the Mediterranean Diet (MD) and the health benefits against youth obesity and associated cardiovascular disease (CVD) risk factors, identifying positive effects exerted by an MD including sourdough bread and healthy products from the Mediterranean basis (chickpeas/hummus, nuts, and pomegranate juice). For this purpose, a multicenter randomized controlled trial in which an MD-based intervention will be compared to a traditional low-fat diet intervention will be carried out with 240 overweight and obese adolescents (13–17 years) from Spain, Portugal, and Italy. Both interventions will be combined with an educational web-application addressed to engage the adolescents through a learning-through-playing approach, using both educational materials and games. To assess the interventions, adherence to the MD, dietary records, physical activity, food frequency, sociodemographic, and quality of life questionnaires as well as classical anthropometric and biochemical parameters will be evaluated. Furthermore, an omics approach will be performed to elucidate whether the interventions can shape the gut microbiota and gut-derived metabolites to gain knowledge on the mechanisms through which the MD can exert its beneficial effects.

## 1. Introduction

According to the World Health Organization (WHO), youth obesity is one of the world’s most challenging public health problems [1,2]. In 2016, 9.9% of children and adolescents between 5 to 19 y were obese, and about one in five children and adolescents (18.7%) was considered overweight in the Organization for Economic Cooperation and Development (OECD) countries [3]. The prevalence of overweight and obesity in children and adolescents is even higher in some Mediterranean countries, the average rates of overweight and obesity being 32.4% in Portugal, 34.1% in Spain, and 36.8% in Italy [3]. This problem needs compelling solutions, considering that adolescent obesity is a strong predictor of adult obesity, which has many well-known short- and long-term health and economic consequences, both for the individual and for the society as a whole.

The Mediterranean Diet (MD) is one of the healthiest diets worldwide. It is characterized by a balanced lipid profile (low in saturated fats (SFAs) and high in monounsaturated fats (MUFAs), with olive oil as the main source of fat), a high intake of low glycaemic index carbohydrates, fiber, antioxidants, cereals (preferably as whole grain), legumes, fruit, vegetables, and nuts, and a moderate consumption of fish and shellfish, white meat, eggs, and dairy products [4,5,6]. Recently, the environmental impact of MD has been addressed and considered in an updated version of the MD Pyramid, which emphasizes a lower consumption of red meat and bovine dairy products, and a higher consumption of legumes and locally grown eco-friendly plant foods [7,8]. Results from the PREDIMED study clearly demonstrated that an MD supplemented with extra-virgin olive oil or nuts was associated with a reduced risk of cardiovascular disease (CVD) and death compared with a low-fat control diet in middle-aged and elderly participants [9,10]. The beneficial effects of the MD on different components of the Metabolic syndrome (MetS), such as abdominal obesity and circulating levels of glucose [11,12], have also been demonstrated. In addition, different studies have shown that MD can also reduce body weight and adiposity, especially when this diet is energy-restricted [13,14]. According to the results of a recent umbrella review of meta-analyses of randomized controlled trials, the Mediterranean diet had the strongest evidence of a beneficial effect on both anthropometric parameters and cardiometabolic risk factors in adult populations [15]. 

Concerning adolescents, some cross-sectional studies showed that a high adherence to the MD was associated with decreased risk of being overweight, obese, and affected by MetS in adolescents from Mediterranean countries [16,17]. However, the health effects of MD, and the underlying mechanisms, have been scarcely evaluated in obese adolescents through well-designed clinical–nutritional intervention studies. Thus, to the best of our knowledge, there was only a pilot RCT, implemented in 49 Mexican obese children (11 years old), which showed that a nutritional intervention based on MD for 16 weeks was more effective in reducing body mass index (BMI) and in improving lipid profile than a standard diet [18]. Therefore, more nutritional intervention studies carried out in large cohorts of adolescents from different Mediterranean countries are needed to shed more light on the beneficial effects of MD against obesity and CVD risk factors in comparison with conventional low-fat diets.

Adherence to MD is easier with respect to the majority of the prescribed hypocaloric diets, which is mainly due to its variety of foods and flavours [5]. Nevertheless, during the last decades, there has been a constant reduction of this adherence among young people in different Mediterranean countries, now ranging from 10% to about 21% [19,20,21]. In addition, European children spend more and more time watching television, playing video games, or on the internet, which implies a decrease in physical activity and energy expenditure, which is a well-known risk factor for obesity and CVD [22,23]. Therefore, it is essential to combine an MD nutritional intervention with the promotion of physical exercise and with educational strategies addressed to increase the will of the adolescents to be compliant with the dietary advice.

A growing body of scientific evidence suggests that the intestinal microbiota plays an important role in maintaining an optimal health status in the host [24,25]. Alterations in the composition of the gut microbiota that lead to a reduction in beneficial bacteria, also known as dysbiosis, may contribute to the pathogenesis of obesity, insulin resistance, type 2 diabetes, and non-alcoholic fatty liver disease (NAFLD) [5,24,25,26]. The gut microbiota also determines how different key nutrients and bioactive molecules, such as bile acids, lipopolysaccharide (LPS), and short-chain fatty acids (SCFAs), exert their signaling actions [27,28]. Some data suggest that MD, or some of its bioactive ingredients, can positively modulate gut microbiota by influencing its composition [29,30,31,32,33], increasing the abundance of beneficial bacteria such as *Lactobacillus*, *Prevotella*, *Bifidobacteria*, and *Enterococcus,* and decreasing the amount of *Clostridium* and *Bacteroides* [30]. In a pilot study, the adherence to a Mediterranean-inspired anti-inflammatory diet slightly normalized the gut microbiota in individuals with Crohn’s disease [31]. Another study showed that high adherence to MD was associated with increased fecal SCFA concentration, as well as decreased levels of urinary trimethylamine N-oxide, which is highly associated with intestinal dysbiosis and cardiovascular risk [34]. However, as far as we know, how a MD-based nutritional intervention can shape the gut microbiota and modulate the production of gut-derived metabolites, as well as the mechanisms by which MD exert its effects on the intestinal bacterial communities and on human health, is far from being understood, and it has not been yet evaluated in adolescent populations.

All the above commented aspects prompted us to build a multidisciplinary consortium of six partners from five Mediterranean countries, with complementary expertise and resources within the areas of nutrition, biochemistry, ICT/Bioinformatics, omics sciences, and food manufacturing, which was created to implement a strategy to tackle youth obesity (MED4Youth project; https://med4youth.eu/ (accessed on 12 April 2021)). This consortium is constituted by four research institutions: Fundació Eurecat (Reus, Spain), University of Coimbra (UC, Coimbra, Portugal), Università di Parma (UNIPR, Parma, Italy) and Scientific Food Center (Amman, Jordan), and two small and medium enterprises (SMEs), NOVAPAN S.L. (Spain) and Shikma Field Crops (Israel). The MED4Youth project arises with the main purpose of assessing whether an energy-restricted MD intervention including healthy and palatable products from the Mediterranean basin (mixed nuts [10], pomegranate [35,36,37], and hummus [38]) and a multigrain sourdough bread with proven health effects [39] is more effective against youth obesity and associated CVD risk factors than a conventional low-fat diet. To achieve this goal, a multicentric nutritional and clinical intervention study, specifically targeting obese/overweight adolescents (13–17 years) from Spain, Italy, and Portugal will be carried out. The study will include an educational approach based on a web-application designed to encourage healthy behaviors. Furthermore, the project will also include an omics and a system biology approach to elucidate whether the MD can shape the gut microbiota and gut-derived metabolites, to shed more light on the mechanisms by which MD may exert its beneficial effects against youth obesity and CVD risk factors.

## 2. Materials and Methods

### 2.1. Study Design and Setting

**Figure 1:**
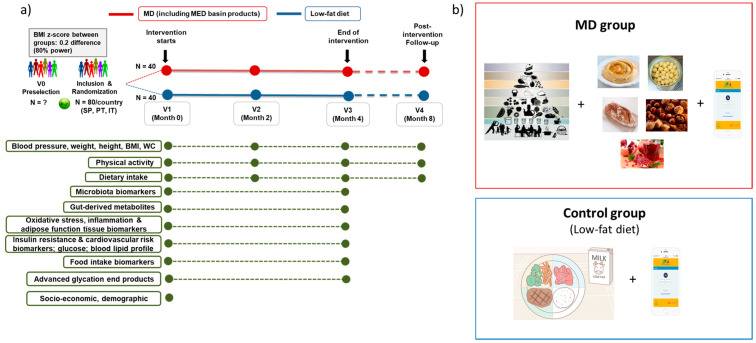
(**a**) Design of the Study. (**b**) Overview of the intervention groups. MD, Mediterranean Diet; MED, Mediterranean; BMI, Body Mass Index; ES, Spain; PT, Portugal; IT, Italy; V, Visit; WC, Waist Circumference.

The overall design of the study is represented in Figure 1. The MED4Youth study is a multicenter single-blinded randomized controlled clinical trial with obese and overweight adolescents (13–17 y) from Italy, Portugal, and Spain, in which an MD-based intervention will be compared to a traditional low-fat diet intervention. The three centers involved in the study obtained the approval of the Ethics Committee of each institution: (1) Eurecat (study coordinator): Institut d’Investigació Sanitària Pere i Virgili (Ref. CEIM: 210/2020); (2) UNIPR: Area Vasta Emilia Nord (AVEN) Ethical Committee (pre-approval Ref. Prot. 28638); (3) UC: Ethics Committee of Centro Hospitalar e Universitário de Coimbra and Portuguese National Data Protection Commission (Ref. CHUC-197-20). MED4Youth study was registered at ClinicalTrials.gov (NCT04719052). The protocol conformed to the Helsinki Declaration and Good Clinical Practice guides of the International Conference of Harmonization. This study followed the CONSORT criteria.

According to the sample size calculations, a total of 240 volunteers from Italy, Portugal, and Spain will be recruited for this study (80 participants per country).

The Spanish subjects will be recruited by Eurecat in the province of Tarragona, the Portuguese subjects will be recruited by the Hospital Pediátrico de Coimbra, and the Italian subjects will be recruited by the Pediatric Clinic of the Maternal and Child Department of the Parma University Hospital. The 80 individuals recruited in each country will be randomly assigned to either a MD group (n = 120, n = 40 per country), which will be instructed to follow an MD that will incorporate sourdough bread, hummus, pomegranate, and mixed nuts, or a Control group (n = 120, n = 40 per country), which will be assigned to a standard low-fat diet. The intervention duration is 4 months for each participant, including 3 visits. A follow-up visit after 4 months from the end of the intervention will be carried out.

The randomization code will be computer-generated using software (Geoffrey C. Urbaniak and Scott Plous, Lancaster, PA, USA). Furthermore, the unit responsible for the randomization will be separate from the study researchers.

For ethical reasons, a control group with no treatment against obesity will not be included, since a meta-analysis of randomized controlled trials for the treatment of overweight in children clearly demonstrates that control groups, whether wait listed or only given written information, consistently gained weight (2.1% increase in percentage overweight post treatment and 2.7% post follow-up) [40]. Due to the nature of the study, the participants cannot be blinded to the intervention, although the investigators who will perform the sample and data analysis will be.

### 2.2. Selection Criteria

Subjects included in the study:

- Boys and girls aged 13–17 years,
- Having obesity, defined as an age- and sex-specific BMI in the 95th percentile or greater (1), or great overweight (age- and sex-specific BMI in the ≥90th to <95th percentile),
- If they and their parents (or holders of parental responsibility over the child) provide written informed consent (signed by parents and by the adolescent), and
- Having easily access to internet (e.g., via mobile phone, tablet, pc).

Exclusion criteria are:

- Having diabetes and other metabolic, endocrine, and chronic disorders,
- Intake of antibiotics, drug, probiotics, or nutritional supplements in the last month,
- Prescribed medicine to control hypertension, inflammation, or dyslipidaemia,
- Following a prescribed diet for any reason, including weight loss, in the last 3 months,
- Following a religion-restricted diet, and
- Having allergies or food intolerances to nuts, pomegranate, bread, and/or chickpeas.

All volunteers will be examined by physician to assess whether they meet the conditions for inclusion. For this purpose, a medical interview, a physical examination, and laboratory tests will be performed.

### 2.3. Dietary Interventions

Both diets (MD and low-fat diet) will be implemented by dietitians/nutritionists from each center. First of all, the total energy requirements of adolescents will be calculated by using the Molnar equation [41], and the level of physical activity will be obtained from the validated Physical Activity Questionnaire for Adolescents (PAQ-A). Afterwards, a caloric restriction of 20% will be applied to design a personalized diet for each obese individual (BMI ≥95th percentile) [42], while caloric restriction will be carefully evaluated for each individual with great overweight (BMI ≥90 to <95th percentile). For ethical issues, adolescents will not be conscious about caloric restriction of their diet, but only about dietary recommendations to follow the specific diet. In addition to the volunteer’s energy requirements, diets will take into account their food preferences and culture.

A weekly meal general plan, including portion sizes and a list of food equivalents will be provided to participants in both groups, so they could adapt and personalize their menus. The dietary prescription will be also easily accessible by both adolescents and their parents through an educational web-application developed by UNIPR partner. In addition, parents will be provided with a daily recipe to facilitate the adherence to the dietary prescription as well as to increase their culinary skills and the engagement in the project. Moreover, in each country, some recipes will be traditional of another country, i.e., a Spanish recipe in Italy or an Italian plate in Portugal.

(1) The MD will be based on a high consumption of unsaturated fat from vegetable sources (extra virgin olive oil and nuts) and minimally processed plant foods (vegetables, fruits, nuts, whole grains and legumes), low consumption of meat (especially red and processed meats) and sweets, and moderate consumption of fish and dairy products (mainly yogurt and cheese) [6,43]. Accordingly, this diet will provide a high amount of mono and polyunsaturated fatty acids, fiber, and phenolic compounds [44]. The MD will be based on the one proposed as MD in the PREDIMED study [45], providing 40–45% of energy from carbohydrate, 35–40% from fat, and 15–20% from protein. Special attention will be paid to the benefits of whole and minimally processed foods, traditional food vs. fast food, and seasonal products. Within the MD pattern, the intervention group (MD) will consume a sourdough bread and 3 specific foods from the Mediterranean basin with proven healthy effects (pomegranate juice, chickpeas/hummus, and mixed nuts) (Table 1).

In summary, adolescents in the MD group will replace the intake of conventional bread by sourdough bread consumption (2 servings of 50–60 g daily), they will incorporate into their diet chickpeas (2 servings of 150 g/week chickpeas, a minimum of one of them being hummus), while they will consume at least another serving of legumes, which can be chickpeas or another legume, and will consume pomegranate juice (4 servings of 200 mL/week) and mixed nuts (4 servings of 30 g/week). The sourdough bread will be a multigrain leavened with Rebola sourdough, which has been previously shown to elicit a lower increase in postprandial blood glucose levels and to decrease the inflammatory marker MCP-1 in rats when compared to a conventional white bread [35]. The squeezed pomegranate will be a single-strength juice. It will be squeezed with the arils, seeds, and inner and outer shell in order to get the important polyphenols existing in the fruit. Chickpeas are a popular pulse rich in protein and dietary fiber and will be consumed boiled and as hummus (paste of chickpeas, tahini, olive oil, lemon, and herbs).

(2) The low-fat diet (control diet) consists of a restricted consumption of fats. This diet will be based on the one proposed as low-fat diet in the PREDIMED study [45], and it will provide 55% of energy from carbohydrate, 25–30% from fat, and 15–20% from protein. The low-fat diet is the most used diet for obesity treatment in adolescents [42]. This group will not receive any additional specific food by the researchers.

Volunteer’s compliance with the dietary prescription will be assessed by means of self-reported weekly food checklists, 3-day dietary records, and selected biomarkers of food intake.

### 2.4. Implementation of the Motivational Interview Technique

Before beginning the intervention, a personalized dietary advice (30-min session) to each participant including recommendations on the desired frequency of intake of specific foods and general recommendations about performing physical activity will be delivered by dietitians/nutritionists by using the psychological “motivational Interview” technique [48]. The motivational interview will have a standardized internal protocol that will follow the dietitians/nutritionists of the 3 each enrolment centers.

In the first session, the attendance of parents, mentors, or caregivers will be recommended, as they can strongly encourage their children and help the adolescents understand and follow the lifestyle recommendations. Especially, parents can help with cooking and with food purchase.

### 2.5. Educational Web-Application

The adolescents of both groups and their parents will have access to an educational web-application to be used during the intervention, where they can learn about health, nutrition, and food in general, how the right food choices positively impact on health, in addition to providing information on sustainability issues and the link between health and sustainability. The application will be developed as a cross-browser web-application with responsive design, so it will be available for both desktop and mobile devices. The application will be accessible at a specified secure URL and will require a username and a password to enter in the main page. The URL and the personal username and password will be provided to each participant at the beginning of the intervention period. Internet will be needed to use the application, independently if they use a computer, a smartphone, or a tablet. To ensure the validation and usability of the web-application, a pilot study will be performed in Italy with teenagers aged 13–17 years. The web-application will be intended for two final user typologies, namely the adolescent participant user and the parent user. For each one of the two users, specific contents will be available on the basis of the intervention group to which they will be assigned (MD or control group). The application will be designed to be used independently in Spanish, Italian, and Portuguese.

For the adolescents allocated to the MD group, the application will also include specific contents about MD, including information about pomegranate juice, hummus/chickpeas, mixed nuts, and sourdough bread.

To help engage participants, a “learning-through-playing” approach will be used, combining together educational materials and games [49], such as group-specific educational contents, group-specific tips of the day, group-specific quizzes, and group-specific recipes of the day. Among the group-specific educational contents (MD or control diet), one topic will be available per each intervention week (16 weeks) with weekly release. In addition, a “welcome” content will be also available at the beginning of the study. Based on the topic of the week, the quiz game will be composed by 15 multiple-choice questions per week: 2 questions per day plus one final general question. The user should answer the right option to move to the next question/level (only two attempts will be available for each question per day), and every week, a new level will be available. To increase participants’ engagement, a scheme with badges/achievements will be available. Adolescents will have access to the application every day to get the “tip of the day”, which is a short and easy “take-home message” defined to improve their food literacy and play with the quiz game. In addition, the application will be used to deliver the personalized dietary plan designed by a dietitian/nutritionist and to collect data during the intervention study by means of 3-day food diaries and questionnaires. Information on healthy diet and nutrition will also be provided to the parents through the app to help them support their kids. In addition, parents will be able to browse the “recipe of the day” to find everyday a new recipe helping them to prepare tasty and nutritious meals, and they will also have access to the weekly educational contents and to the diet of their children.

### 2.6. Manufacturing and Distribution of the Mediterranean Basin Foods

The sourdough bread will be manufactured by NOVAPAN. Afterwards, NOVAPAN will distribute the sourdough bread to the three enrollment centers. These breads will be sent several times to each institution during the study, will be stored at the centers at −20 °C, and will be provided periodically by the researchers of each center to the adolescents (and/or parents of the adolescents) allocated in the MD group. The breads will be provided to the volunteers at no cost, and an extra amount of breads will be given for the rest of family in order to increase compliance.

Chickpeas/hummus and pomegranate juice will be sent by Shikma Field Crops to each center and will be provided to the MD group. Chickpeas/hummus and pomegranate will be sent several times during the study. Hummus and pomegranate will be delivered in refrigerated conditions, while chickpeas will be delivered cooked and canned.

Walnuts, almonds, and hazelnuts will be distributed by the company COSELVA (La Selva del Camp, Tarragona, Spain) to the different centers. The nuts will be packaged by COSELVA in an individual package containing 10 g for each nut (walnuts, almonds, and hazelnuts).

Moreover, extra virgin olive oil (EVOO) will be provided to the MD group in order to ensure that this group uses this type of fat for cooking and dressing. EVOO will be distributed by COSELVA to the different centers.

### 2.7. Outcomes

The primary outcome is the BMI z-score, which is a standardized measure of BMI based on specific age and sex [50,51]. The effectiveness of the intervention will be evaluated by comparing the BMI z-score between the control (low-fat diet) and intervention (MD) groups.

The secondary outcomes are:

- MD adherence, using the KidMed questionnaire [19]
- Physical activity, using Physical Activity Questionnaire for Adolescents (PAQ-A) [52]
- Dietary intake and habits:
  ○ 3-day dietary records [53]
  ○ Food frequency questionnaire (FFQ) of the Helena study [53]
- Nutritional knowledge, using the NK Helena questionnaire [54]
- Quality of life, using the kidscreen-27 Index [55]
- Sociodemographic data, using the Health Behavior in School Age Children (HBSC) socio-demographic questionnaire [56]
- Anthropometric data: weight, height, BMI (weight/height^2^), body composition using bioimpedance, waist circumference (WC), and waist–hip ratio (WHR)
- Blood pressure
- Biochemical variables, gut microbiota composition, and metabolomics in urine, blood, and feces

All outcomes are presented and summarized in Table 2.

The questionnaires will be used to collect data on the habits and knowledge of the participants in different visits. These data will be collected through the app and then combined with anthropometric and food intake data to assess possible associations among these variables.

At visit (V)1, V2, V3, and V4, the subjects will attend the partner center in over-night fasting conditions to evaluate anthropometric parameters, dietary intake, physical activity, and blood pressure. At V1 and V3, feces, blood, and morning urine will be collected to perform the analyses of microbiota and gut-derived metabolites; food intake biomarkers and phenolic metabolites; circulating levels of glucose and blood lipid profile, biomarkers of insulin resistance, adipose tissue function, inflammation, oxidative stress, and cardiovascular risk, and advanced glycation end products (AGEs). In addition, the lipid profile of the erythrocyte membrane will be assessed at V1 and V3. Data collected at V4 will be used to investigate if the intervention will have changed medium-term dietary habits, having a medium-term impact also on blood pressure and anthropometric parameters. Additionally, for female adolescents, information about their menstrual cycle will be recorded in each visit. Table 3 shows the schedule of each visit, which will follow the same timeline for each enrollment center.

### 2.8. Sample Size

According to the sample size calculations based on differences in BMI z-score (primary outcome) between groups (0.2 difference with 80% power; two-tailed α: 0.05; standard deviation: 0.5; dropout rate: 20%), a total of 240 volunteers from three Mediterranean countries (Spanish, Portugal and Italy) will be recruited for this study (80 participants per country) after obtaining the approval of the Ethics Committees of each recruiting center and the written informed consent from parents and from subjects.

### 2.9. Statistical Analyses

#### 2.9.1. Univariate Data Analysis

Data will be expressed as mean ± standard deviation (SD). Grubbs’ test will be used to detect outliers, which will be discarded before subsequent analyses. The normality of variables will be assessed by the Kolmogorov–Smirnov test. Non-parametric variables will be log transformed. Differences in baseline characteristics between subject groups will be analyzed by analysis of covariance (ANCOVA), in which dietary intervention group assignment (G) will be used as a fixed factor and the variables age and sex will be included as covariates. Analyses will be made by intention-to-treat. An ANCOVA model will be used to analyze the data at the end point. The anthropometric and biochemical parameters (changes from baseline) will be used as dependent variables, dietary intervention (MD; low-fat diet) will be included as fixed factor, and the variables age and sex, as well as baseline values, will be included as covariates. Within each group (MD and low-fat diet), changes from baseline will also be analyzed at the end point by a paired *t* test. Within each group (MD and low-fat diet), changes from baseline will also be analyzed considering the different time points using a general linear model for repeated measures. All statistical analyses will be performed with SPSS Statistics (SPSS, Inc., Chicago, IL, USA), setting the level of statistical significance at bilateral 5%. For metagenomics analysis, the non-parametric Mann–Whitney U test will be performed to elucidate pairwise differences on bacteria relative abundance among dietary groups. The Benjamini–Hochberg method will be used to adjust P values for multiple testing considering a 5% false discovery rate. Statistical analyses will be conducted with open-access STAMP software.

#### 2.9.2. Multivariate Data Analysis

For the NMR metabolomics analyses in feces, multivariate modeling will be performed in MATLAB (Mathworks Inc., Natick, MA, USA), using in-house scripts as previously described [57]. Briefly, it will firstly include a principal component analysis (PCA) to identify clusters and outliers, followed by pairwise comparisons between dietary groups using an orthogonal projection to latent structures discriminant analysis (OPLS-DA) to identify discriminatory metabolites. Colors projected onto an OPLS coefficient plot will indicate the correlation coefficient (r2) between each metabolite and grouping variable, with red indicating strong significance and blue indicating weak significance.

For the metabolomics and metagenomics data, unsupervised hierarchical clustering analysis (HCA) will be performed to identify general patterns of gut-derived metabolites or gut microbiota variations between samples, based on metabolites identified in the OPLS-DA models or bacterial genera present in at least the 5% of the samples and with ≥1% abundance. Data will be standardized as z-scores across samples for each metabolite and microbial genera before clustering so that the mean will be 0 and the SD will be 1.

For clustering identification, a multivariate approach based on PCA, partial least squares discriminant analysis (PLS-DA) and HCA, including the anthropometric and biochemical parameters, food intake biomarkers and phenolic metabolites, gut-derived metabolites, and bacterial genus abundances will be carried out after data normalization and autoscaling.

#### 2.9.3. Data Integration: Systems Biology Approach

The systems biology approach aims to integrate the multiple layers of information generated in the course of the study (classical anthropometric and biochemical biomarkers of health, AGEs, dietary and physical habits, social aspects, food intake biomarkers and omics data (gut microbiota and gut-derived metabolites) to extract conclusions from a holistic point of view. The system biology approach will allow gaining a detailed understanding of the mechanisms by which the MD intervention exert its effects and to identify a combination of biomarkers that accurately characterizes the changes induced by the MD intervention. The integration of the anthropometric and biochemical data, metagenomics, and metabolomics will be based on mixDIABLO (http://mixomics.org/mixdiablo/ (accessed on 20 December 2020)), which is an integrative method to identify multi-omics biomarker panels that can discriminate between multiple phenotypic groups. DIABLO uses PLS and extends both sparse PLS-DA to multi-omics analyses and sparse Generalized Canonical Correlation Analysis to a supervised analysis framework [58].

## 3. Discussion

The identification of lifestyle factors, including adoption of dietary patterns, to counteract the troubling youth obesity epidemic, is of primary importance. However, it is still unclear which strategies or combination of strategies are effective in different contexts across the globe for obesity prevention in young people. In this framework, the MED4Youth project will develop, for the first time, a multicenter nutritional intervention study to evaluate the health benefits of the MD against youth obesity and associated CVD risk factors.

The novelty of the MED4Youth project relies on, firstly, carrying out a multicenter clinical and nutritional intervention study for 4 months, including ICT educational tools, specifically targeting 240 obese adolescents (13–17 years) from different Mediterranean countries (Spain, Portugal, and Italy), and, secondly, the application of omics technologies and a systems biology approach to elucidate whether the MD could shape the gut microbiota and gut-derived metabolites, trying to pinpoint the mechanisms by which MD exert its beneficial effects against youth obesity and CVD risk factors.

Moreover, the MED4Youth project will contribute to valorizing Mediterranean products, especially foods not widely consumed in European Mediterranean developed countries (sourdough bread, hummus, and pomegranate), promoting their consumption in the overall population, especially in adolescents, and paving the way for a high, long-term demand of these products, fostering the competitiveness of processing industries and reinforcing local production systems.

Another strong point of this intervention study is that subjective, conventional dietary assessment tools will be combined with targeted metabolomics analyses of selected food intake biomarkers to provide a more accurate fingerprinting of individuals’ dietary pattern. This will allow establishing robust associations between MD adherence and health status. In addition, the analysis of the inter-individual differences associated with particular biomarkers of intake of some plant products and phenolic classes will serve to assess the true internal exposure to certain bioactive compounds. This personalized approach will represent a step forward toward improving dietary exposure evaluation, allowing a more objective and robust assessment of the dietary pattern of the adolescent.

On the other hand, the multidisciplinary consortium will allow exchanging best practices among Mediterranean basin countries regarding adherence to MD, creating a common knowledge and understanding on how MD can be effective toward preventing youth obesity, fostering long-lasting results and supporting policies to prevent and ameliorate obesity across the Mediterranean basin.

MD is characterized by a high palatability, the use of herbs and spices, and the election of traditional culinary techniques, and this will facilitate adherence to the intervention and will increase long-term compliance, in contrast to most of the low-calorie diets prescribed these days, which are less tasty and difficult to follow, especially in the long term. However, there is a low adherence to MD among the youth population of different Mediterranean countries, with around 10% of adolescents showing a medium–high adherence and 21% present a low adherence to this diet (low adherence 0–3 points/12; medium adherence 4–7 points/12; high adherence: 8–12 points/12) [20,21]. Therefore, there is a clear interest in identifying palatable healthy foods to try to increase the adherence to MD. Thus, in the present project, some traditional Mediterranean foods are proposed to the volunteers of all the centers, which are all characterized by a healthy nutrient profile and still very palatable, namely nuts, chickpeas/hummus, and sourdough bread. In particular, the bread will be made with flours from ancestral cereals and cereals with a lower gluten content, with a higher fiber content, and a lower carbohydrate content than conventional fast-fermenting bread made with white wheat. The inclusion of sourdough bread is based on previous scientific research that showed improved nutritional quality and beneficial effects on health with respect to the use of conventional breads, through the induction of satiety, a better control of the glycemic and insulinemic responses, the attenuation of systemic inflammation, and a favourable modulation of the gut microbiota [59,60,61,62]. Nuts, hummus/chickpeas, and pomegranate juice are rich sources of different bioactive compounds with putative cardioprotective effects that can contribute to reducing obesity, such as MUFA, PUFA, fiber, vitamins E and C, and different phenolic compounds [35,63,64,65]. There is strong evidence pointing towards a beneficial impact of nuts consumption on different cardiovascular risk factors, while their intake has been reported to help in the process of weight loss [66]. 

Moreover, different studies suggest that hummus and chickpeas may play a beneficial role in weight management and glucose control, as well as have a positive impact on some markers of CVD [38,63]. Finally, pomegranate and pomegranate juice are a rich source of polyphenolic compounds, which have shown several beneficial effects on human health [67], could positively impact the gut microbiota, and have been shown to contribute the modulation of metabolic disorders, including insulin resistance, which is typically associated to obesity [35,36,37]. Pomegranate juice intake has also been related with improvements on endothelial function in adolescents [68]. In the MED4Youth project, pomegranate will be consumed as pomegranate juice (delivered in frozen form) in order to facilitate and ensure its intake in the volunteers.

Some psychologists emphasize the importance of creating new habits, changing people in their daily context, influencing their habits and their basic psychology, rather than following traditional approaches based on simply providing information and education, to achieve a sustained change in behavior that has a beneficial impact on health [69,70]. Related with this issue, the MED4Youth project targets adolescence (13–17 y) as a key and sensitive development stage of life, where healthy lifestyles and behavioral flexibility need to be reinforced. For this reason, obese adolescents will be provided with an educational web-application where they can learn about health, nutrition, and food in general. Moreover, the motivational interview, using a nonjudgmental, empathetic, and encouraging tone, has shown to favor behavior change in adolescents, including the adoption of healthy dietary habits [44,53]. For this reason, the motivational interview used in the MED4Youth project will be focused on behavioral change, motivation for change, and no confronting.

Some potential limitations need to be taken into account. First, while volunteers in the MD group will receive some foods to be included in their diet, volunteers in the control group will not, so adherence to the low-fat control diet may be lower. Second, due to the nature of the interventions, participants and dietitians/nutritionists cannot be blinded, although researchers who will perform all biochemical and -omics determinations as well as statistical analyses will be. Another limitation is the short follow-up period (4 months) that might limit conclusions on the long-term effects. On the other hand, the major strengths of the study can be listed: (a) this is the first large RCT to evaluate the health benefits of the MD against youth obesity and associated CVD risk factors, with a total of 240 volunteers; (b) this is a multicentric study carried out in three different Mediterranean countries, which facilitates the generalization of the conclusions achieved from this study; (c) adherence in both groups will be fostered through the availability of an educational web-application intended to be used by both parents and adolescents, which provides information related to health and nutrition as well as recipes, and that will allow a “learning-through-playing” approach; (d) the use of food intake biomarkers provides an objective assessment of adherence to dietary interventions and a robust measurement of the dietary pattern of the volunteers; and (e) the application of omics technologies and a systems biology approach, together with the wide range of biomarkers analyzed, will provide a significant progress in the current knowledge on the mechanisms implicated in the beneficial effects of MD as well as the role of gut microbiota in these effects.

## 4. Conclusions

The MED4Youth project aims at determining whether an MD-based intervention including sourdough bread and healthy Mediterranean foods (chickpeas, humus, mixed nuts, and pomegranate juice) is more effective than a standard low-fat diet to tackle youth obesity, by conducting a multicenter randomized controlled trial with 240 overweight and obese adolescents from Spain, Portugal, and Italy. The dietary interventions will also include ICT educational tools. The scientific data derived from this project, especially those generated by the application of omics technologies, are expected to provide a deeper understanding of the mechanisms implicated in the beneficial effects of MD against youth obesity and the associated CVD risks factors, as well as the role of the gut microbiota. The MED4Youth project will help to establish an effective intervention for obese adolescents and therefore will help to prevent adult obesity and its comorbidities. Public health policies for obesity prevention could also be supported by the results of this project.

Further studies on larger cohorts would be crucial for the validation of the health biomarkers analyzed in the MED4Youth project in order to clearly demonstrate the underlying mechanisms behind the effects of MD against youth obesity and the associated risk factors. Moreover, on the basis of the results obtained through the analysis of food intake biomarkers in the present study, future studies could be designed to unravel which specific foods or bioactive compounds present in the MD are involved in the beneficial effects described for this dietary pattern.

## Figures and Tables

**Table 1:** Details on the frequency portions of the Mediterranean basin foods.

Specific Food	Frequency of Portions
Pomegranate	It is recommended at least 2 servings/day of fruit: 14–21 servings/week In addition to the fresh whole fruit, 4 servings of pomegranate juice (200 mL) will be consumed during the week.
Hummus and chickpeas	It is recommended 3 servings/week of pulses (150–200 g (50–80 g dry)). Two servings/week must be cooked chickpeas, minimum one of them as hummus, and another serving may be chickpeas or different pulses. The portion size of hummus is 200 g.
Nuts (almonds, walnuts, and hazelnuts)	It is recommended 3 servings/week: 30 g of mixed nuts/serving. Four servings will be consumed to properly combine the 4 food items included in the MD along the week. Mixed nuts will be walnuts, almonds, and hazelnuts.
Sourdough bread	It is recommended 4–6 servings/day of cereals. Two of these must be a serving of sourdough bread (1 serving of bread is approximately 50–60 g [46,47]).

**Table 2:** Variables to be analyzed.

Variables	Tool	Timeline
Blood pressure, weight, height, BMI, body composition, WC, WHR	Dietitians/Nutritionists	V1, V2, V3, V4
Physical activity	PAQ-A *	V1, V2, V3, V4
Dietary intake	3-day dietary record *	V1, V2, V3, V4
Food habits	FFQ Helena questionnaire *	V1, V2, V3, V4
MD adherence	KidMed questionnaire *	V1, V2, V3, V4
Quality of life	Kidscreen-27 questionnaire *	V1, V2, V3, V4
Sociodemographic data	HBSC questionnaire *	V1
Knowledge on food and nutrition	NK Helena questionnaire *	V1, V3, V4
Microbiota (feces)	16sRNA sequencing	V1 and V3
Gut-derived metabolites (LPS, SCFAs, lactate, bile acids) (feces and/or plasma)	NMR/GC-MS	V1 and V3
Biomarkers of oxidative stress (8-OHdG, F2-isoprostanes) (urine)	ELISA	V1 and V3
Biomarkers of inflammation (IL-6, CRP, TNFα, MCP1, IL-8) (plasma)	Magnetic bead-based multiplex assays Homeostatic Model Assessment for insulin resistance	V1 and V3 V1 and V3
Biomarkers of adipose tissue function (adiponectin, leptin, resistin) (plasma)		
Biomarkers of insulin resistance (plasma)		
Biomarkers of cardiovascular risk (TMAO) (plasma and urine)	UHPLC‑MS	
Circulating levels of glucose and blood lipid profile	Enzymatic assays	V1 and V3
Key food intake biomarkers and phenolic metabolites (urine)	Targeted metabolomics (UHPLC‑MS)	V1 and V3
Advanced glycation end products (AGEs) related analyses (plasma or erythrocytes)	ELISA / enzymatic assays	V1 and V3

V: visit; BMI: body mass index; WC: waist circumference; WHR: waist–hip ratio; MD: Mediterranean diet; LPS: lipopolysaccharides; SCFAs: short-chain fatty acids; IL-6: interleukin 6; CRP: C-reactive protein; TNFa: tumor necrosis factor alpha; MCP1: monocyte chemoattractant protein-1; IL-8: interleukin 8; TMAO: trimethylamine N-oxide; PAQ: physical activity questionnaire; FFQ: food frequency questionnaire; HBSC: health behavior in school-aged children; NKQ: nutrition knowledge questionnaire; NMR: nuclear magnetic resonance; GC-MS: gas chromatography coupled to mass spectrometry; ELISA: enzyme-linked immunosorbent assay; UHPLC-GC ultrahigh-pressure liquid chromatography coupled to mass spectrometry. * These variables will be collected through the app.

**Table 3:** Description of study visits.

Visit	Type of Visit	Description
0	pre-screening visit before randomization	To check inclusion/exclusion criteria in the study
1	after randomization, inclusion visit	Feces, urine, and blood samples will be collected, anthropometric and blood pressure will be measured, and questionnaires (food, knowledge and physical activity) and dietary records will be completed.
2	after 2 months	Anthropometric and blood pressure will be measured, and questionnaires (food, knowledge and physical activity) and dietary records will be completed.
3	after 4 months, final study visit	Feces, urine, and blood samples will be collected, anthropometric and blood pressure will be measured, and questionnaires (food, knowledge and physical activity) and dietary records will be completed.
4	visit 4 months after the end of the intervention	Anthropometric and blood pressure will be measured, and questionnaires (food, knowledge and physical activity) and dietary records will be completed.

## Data Availability

No new data were created or analyzed in this study. Data sharing is not applicable to this article.
